# Supplementary figures and images for: Deciphering the phospho-signature induced by hepatitis B virus in primary human hepatocytes
Source: Front Microbiol. 2024 May 22;15:1415449. doi: 10.3389/fmicb.2024.1415449 (PMC11150682; doi:10.3389/fmicb.2024.1415449)

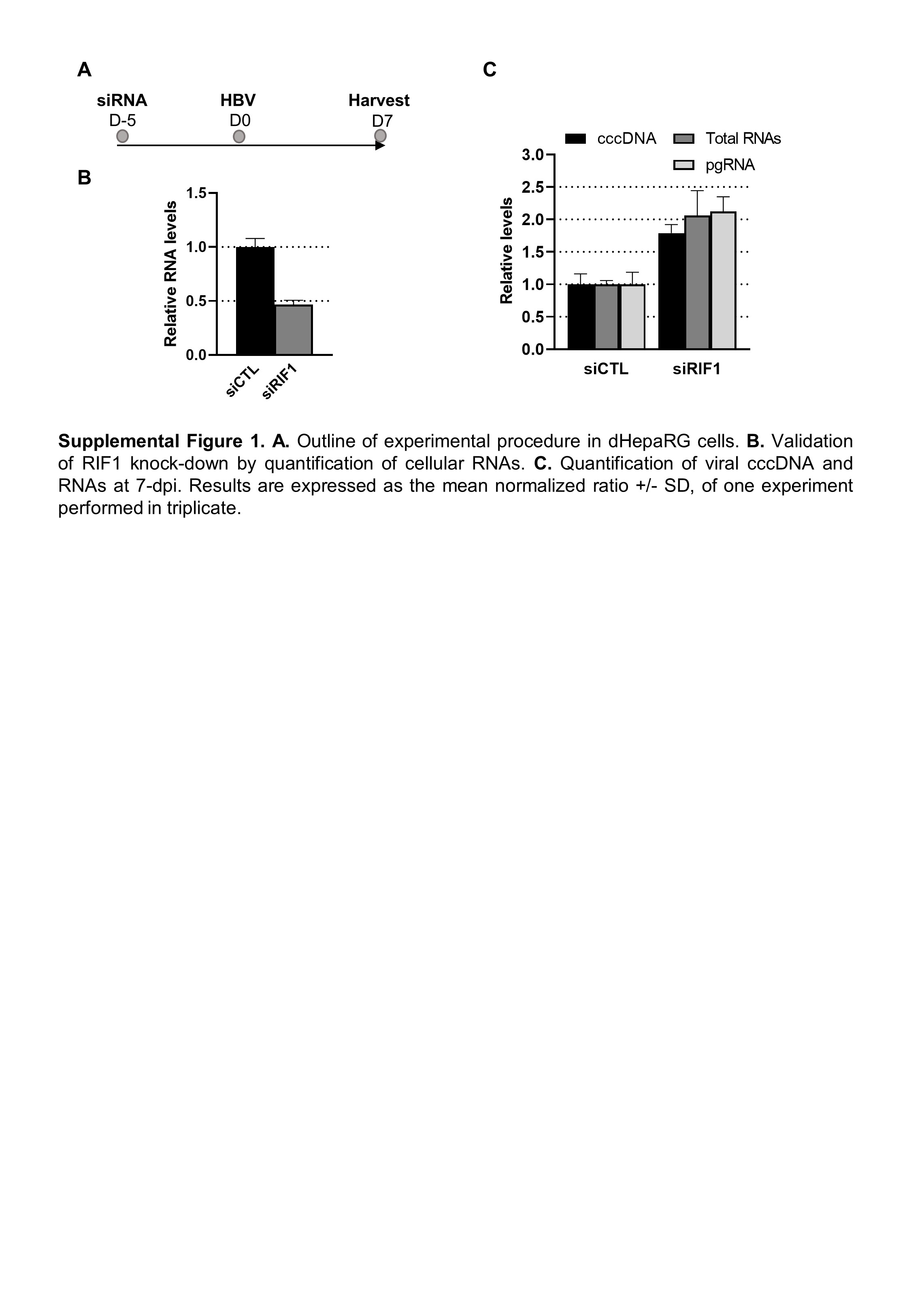

Supplement: Supplementary file 1 [file Image_1.JPEG]

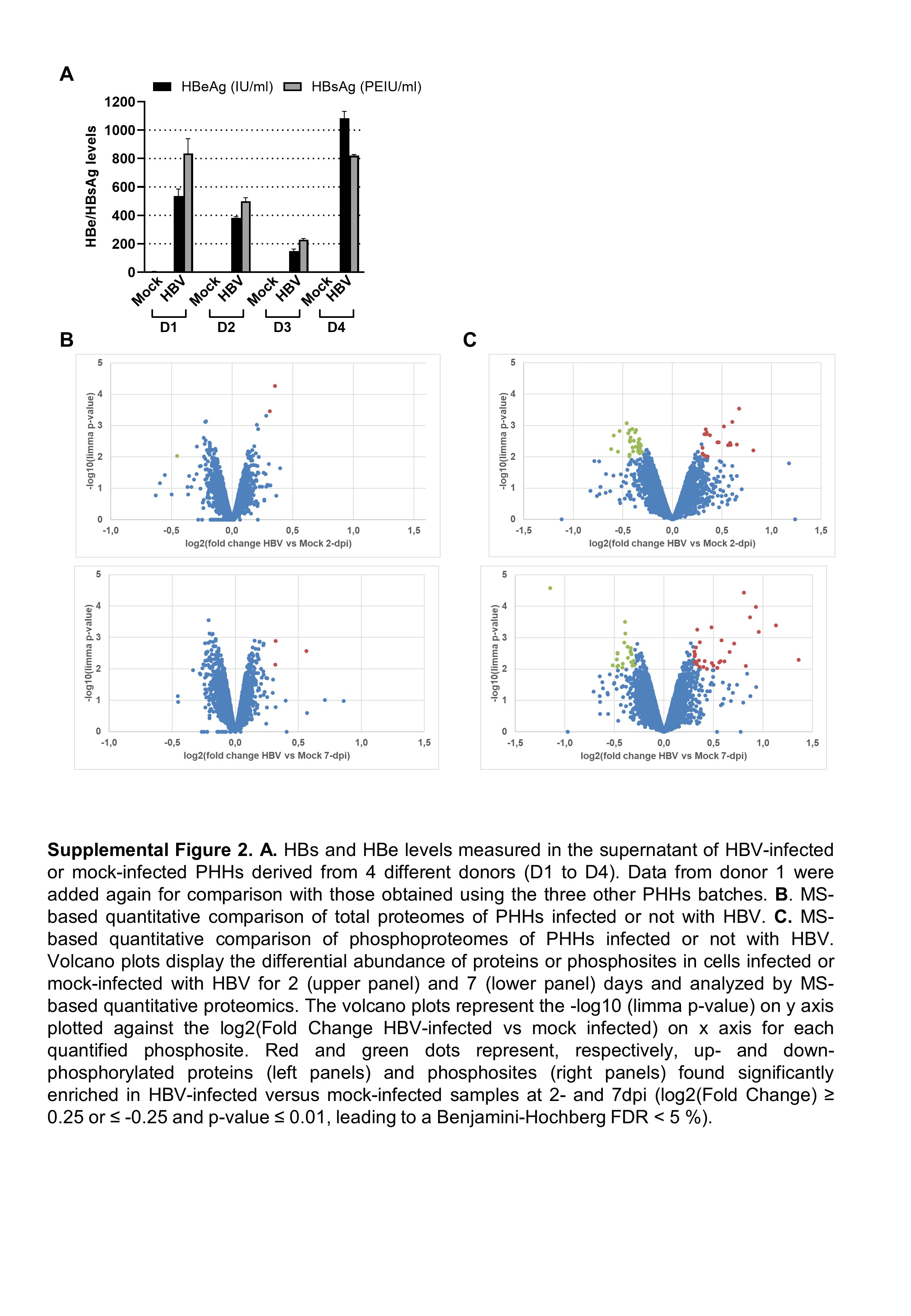

Supplement: Supplementary file 2 [file Image_2.jpg]
